# Elimination of Leprosy as a public health problem by 2000 AD: an epidemiological perspective

**DOI:** 10.4314/pamj.v9i1.71176

**Published:** 2011-05-17

**Authors:** Dickson Shey Nsagha, Elijah Afolabi Bamgboye, Jules Clement Nguedia Assob, Anna Longdoh Njunda, Henri Lucien Foumou Kamga, Anne-Cécile Zoung-Kanyi Bissek, Earnest Nji Tabah, Alain Bankole OO Oyediran, Alfred Kongnyu Njamnshi

**Affiliations:** 1 Department of Public Health and Hygiene, Faculty of Health Sciences, University of Buea, Buea, Cameroon; 2 Department of Epidemiology, Medical Statistics and Environmental Health (Formerly Department of Preventive and Social Medicine), Faculty of Public Health, College of Medicine, University of Ibadan , Ibadan, Nigeria; 3 Department of Biomedical Sciences, Faculty of Health Sciences, University of Buea, Buea, Cameroon; 4 Department of Medical Laboratory Sciences, Faculty of Health Sciences, University of Buea, Buea, Cameroon; 5 Department of Internal Medicine & Specialties (Dermatology and Neurology), Faculty of Medicine and Biomedical Sciences, University of Yaounde ICameroon, Yaounde; 6 Department of Internal Medicine & Specialties (Dermatology and Neurology), Faculty of Medicine and Biomedical Sciences, University of Yaounde I, Yaounde, Cameroon

**Keywords:** Leprosy, elimination, multi-drug therapy, public health, eradication, epidemiology

## Abstract

**Introduction:**

Leprosy is caused by *Mycobacterium leprae* and manifests as damage to the skin and peripheral nerves. The disease is dreaded because it causes deformities, blindness and disfigurement. Worldwide, 2 million people are estimated to be disabled by leprosy. Multidrug therapy is highly effective in curing leprosy, but treating the nerve damage is much more difficult. The World Health Assembly targeted to eliminate leprosy as a public health problem from the world by 2000. The objective of the review was to assess the successes of the leprosy elimination strategy, elimination hurdles and the way forward for leprosy eradication.

**Methods:**

A structured search was used to identify publications on the elimination strategy. The keywords used were leprosy, elimination and 2000. To identify potential publications, we included papers on leprosy elimination monitoring, special action projects for the elimination of leprosy, modified leprosy elimination campaigns, and the Global Alliance to eliminate leprosy from the following principal data bases: Cochrane data base of systematic reviews, PubMed, Medline, EMBASE, and the Leprosy data base. We also scanned reference lists for important citations. Key leprosy journals including WHO publications were also reviewed.

**Results:**

The search identified 63 journal publications on leprosy-related terms that included a form of elimination of which 19 comprehensively tackled the keywords including a book on leprosy elimination. In 1991, the 44th World Health Assembly called for the elimination of leprosy as a public health problem in the world by 2000. Elimination was defined as less than one case of leprosy per 10000-population. Elimination has been made possible by a confluence of several orders of opportunities: the scientific (the natural history of leprosy at the present state of knowledge), technological (multi-drug therapy and the blister pack); political (commitment of governments) and financial (support from NGOs for example the Nippon Foundation that supplies free multi-drug therapy) opportunities. Elimination created the unrealistic expectation that the leprosy problem could be solved by 2000. First, the elimination goal was not feasible in several areas which had high incidence of leprosy. Even if elimination was to be attained, significant numbers of new cases of leprosy would continue to occur and many people with physical imperfections, severe psychological, economic and social problems caused by leprosy would need continuous assistance. Extra-human reservoirs of *Mycobacterium leprae*, the relationship between leprosy and poverty, prevention of disabilities, lack of a reliable laboratory test to detect subclinical infection and a vaccine are also challenging issues.

**Conclusion:**

The evidence base available to inform on leprosy elimination is highly positive with the availability of multi-drug therapy blister packs. There are concerns that leprosy was not the right disease to be targeted for elimination as there are no reliable diagnostic tests to detect subclinical infection including the lack of a vaccine, extra-human reservoirs (monkeys and armadillos), increase in the burden of child cases, no good epidemiological indicator as prevalence instead of incidence is used to measure elimination. Multi-drug therapy treats leprosy very well but there is no proof that it concurrently interrupts transmission. The high social stigma, prevention of disabilities, and the relationship between leprosy and poverty are still major concerns.

## Introduction

Leprosy is caused by *Mycobacterium leprae* and manifests as damage to the skin and peripheral nerves. The disease is dreaded because of the damage that occurs in weak and anaesthetic hands and feet, as well as in blindness and facial disfigurement [1]. Worldwide, 2 million people are estimated to be disabled by the consequences of leprosy. Multidrug therapy (MDT) is highly effective in curing the mycobacterial infection, but treating the nerve damage is much more difficult [1]. In 1991, the 44th World Health Assembly set a target for the elimination of leprosy from the world as a public health problem by 2000 [2]. Elimination was defined as a prevalence of less than 1 case per 10 000 population. Many people found this definition difficult to understand. The "elimination of leprosy" slogan galvanized activities worldwide but has also dominated the priorities in leprosy work [1].

By the end of 2000, 108 of the 122 countries originally listed as leprosy endemic by the WHO, attained the elimination goal at the national level. By the end of 2005, 116 of the 122 leprosy endemic countries had attained the goal. Extra efforts were still needed to achieve the elimination goal at the national level in the remaining six countries. The elimination strategy is based on detecting and treating all leprosy cases with MDT and thereby reducing the disease burden to a very low level. The key is to ensure that all new cases continue to have access to MDT services [3]. MDT is based on the combination of dapsone, rifampicin, and clofazimine which was introduced in 1982 after dapsone-resistant strains appeared and spread. MDT proved highly efficacious in killing the bacteria without inducing resistance, although the optimal length of treatment and associated relapse rates are still controversial [4]. With such a powerful weapon at hand, a massive international effort was launched to eliminate leprosy worldwide [5].

Eradication would mean the complete absence of leprosy and the organism (*Mycobacterium leprae*) that causes it throughout the world. At present, we lack the tools both to protect people from developing leprosy and to diagnose and treat the disease in its sub-clinical form. Significant resources would be required to develop and deploy the necessary tools, and hence, it was decided to aim at elimination of the disease as a public health problem, as the first step [3] instead of eradication. The diagnosis and treatment of leprosy with MDT remain key elements in the strategy to eliminate the disease as a public health problem. MDT treatment has been made available by the WHO free of charge to all patients worldwide since 1995, and provides a simple and yet highly effective cure for all types of leprosy [6].

The global target of eliminating leprosy was reached in 2000 with world prevalence of less than 600,000 cases [4]. According to official reports received during 2010 from 141 endemic countries and territories, the global registered prevalence of leprosy at the beginning of 2010 stood at 211,903 cases, while the number of new cases detected during 2009 was 244,796 [6]. In 2009, the highest prevalence was recorded in South America (2cases and above per 10000) and Africa (1-2 cases per 10000). No cases were reported from Mediterranean Africa and no data was available for Europe, Canada and the USA [7].

The regional leprosy prevalence in the South East Asian region declined from 4.6/10000 population in 1996 to 1.05/10000 population in 2005 [8]. The regional new case detection declined from a peak of 47.8/100000 population in 1998 to 17.9/100000 population in 2004. The SEA Region was on the verge of achieving the leprosy elimination goal at the regional level and in countries, by the end of 2005. Among the 11 countries of the Region, India, Nepal and Timor-Leste were yet to achieve elimination, with prevalence of 1.2, 1.8 and 3.9/10000 population respectively in 2005 [8]. The remaining eight countries had achieved and sustained the elimination goal at the national level. The prevalence and new case detection rate declined in all countries in 2004 compared to 2003, except in Indonesia, where the annual new case detection rate remained static around 15000 cases. The decline was most significant in India with 44% reduction in prevalence and 29% decline in new case detections, due to vigorous efforts in minimizing ‘operational factors’ which were influencing the indicators previously [8].

In Nigeria, new leprosy cases detected annually declined from 226 cases in 2004 to 140 cases in 2008. The prevalence has remained between 0.3-0.4 per 10,000 population within the last five years period. Child proportion among new cases dropped from 12% in 2004 to 5% in 2007 and increased to 9% in 2008 and grade 2 disability among new cases has remained very high between 21%-27% within the previous five years period [9].

Most previously highly endemic countries have now reached elimination. During 2007, both the Democratic Republic of Congo and Mozambique reached this important stage. Pockets of high endemicity still remain in some areas of Angola, Brazil, Central African Republic, Democratic Republic of Congo, India, Madagascar, Mozambique, Nepal, and the United Republic of Tanzania. However, these countries remain highly committed to eliminating the disease, and continue to intensify their leprosy control activities [6].

After the creation of the Global Alliance to Eliminate Leprosy in November 1999 and the drafting of the WHO's “Final Push” strategy (2000-2005) to eliminate leprosy, many partners supported the elimination struggle including the WHO, the World Bank, the International Leprosy Federation (ILEP), the Nippon Foundation and the Sasakawa Memorial Health Foundation (SMHF), Novartis, the Danish International Development Agency (DANIDA) and many more [10]. Even with the global partnership, there are still hurdles to the elimination struggle such as lack of a reliable laboratory test to detect subclinical infection, no vaccine, extra-human reservoirs of *Mycobacterium leprae*, high social stigma and political upheavals in some countries. The objective of this study was to assess the successes of the leprosy elimination strategy, elimination hurdles and the way forward for leprosy eradication.

## Methods

We used a structured search to identify publications on the elimination strategy. The keywords used were leprosy, elimination and 2000. The search identified 63 journal publications on leprosy-related terms that included a form of elimination of which 19 comprehensively tackled the keywords including a book on leprosy elimination. To identify potential elimination publications, we included papers on leprosy elimination monitoring, special action projects for the elimination of leprosy, modified leprosy elimination campaigns, and the Global Alliance to Eliminate Leprosy from all countries of the world. We searched the following principal data bases: Cochrane data base of systematic reviews, PubMed, Medline, EMBASE, and the Leprosy data base.

The following key journals were reviewed: International Journal of Leprosy and other Mycobacteria Diseases, Leprosy Review, The Lancet, PLOS Medicine, WHO publications, International Journal of Epidemiology, World Health Forum, American Journal of Public Health and Hygiene, Indian Journal of Dermatology, venerology and leprology, Journal of Public Health in Africa, Nigerian Quarterly Journal of Hospital Medicine, ILEP publications, Bulletin of the WHO, and Epidemiology and Infection. Data from books, reports from ministries of health and conference/workshop proceedings were also reviewed. We also accessed and retrieved data from websites such as http://www.who.int/lep, http://www.searo.who.int, http://www.paho.org, http://www.Ilep.org.uk. Reference lists for important publications were also scanned.

## Results and Discussion

### Progress towards elimination

The search identified 63 journal publications on leprosy-related terms that included a form of elimination of which 19 comprehensively tackled the keywords including a book on leprosy elimination. The use of MDT, first recommended by the WHO in 1982 led to significant reductions in leprosy prevalence in several countries [11]. In 1991, when the estimated number of leprosy cases around the world had come down to 5.5 million because of the MDT implementation, the 44th World Health Assembly called for the elimination of this disease by 2000 which was defined as less than one case of leprosy per 10.000 inhabitants [12]. To help maintain the target of leprosy elimination, a special working group was established with members from all six regions of the WHO and representations from non-governmental organisations concerned with leprosy [13]. Support for control programmes was forthcoming from various sources. Leprosy control was supported particularly by non-governmental organisations and the Nippon Foundation pledged to meet substantial needs in leprosy drugs until 2000 [14].

As a result of the new strategy, the estimated number of leprosy cases in the world dropped to 3.1 million in 1993 (prevalence of 56.1 cases per 10000) and 2.4 million in 1994 (prevalence of 4 cases per 10000). Reductions were observed in all endemic regions including Africa despite political upheavals and economic difficulties [15].

In mid-1998, more than 8.5 million people had been cured of leprosy using MDT, the dosage and duration of treatment being tailored to the severity of the infection. While in 1985 there were 122 countries with a prevalence of more than one case per 10.000 population, in 1988 there were 55 countries striving to reach the target of elimination [15]. Elimination has been made possible by the confluence of several orders of opportunities: not only the scientific opportunity (the natural history of the disease at the present state of knowledge) but also technological (MDT and the blister pack); political (commitment of governments) and financial (support from NGOs, for example, the Nippon Foundation supplied MDT) [16]. Under the leadership of WHO, full use was made of these opportunities. Objectives were defined in terms of prevalence and time frames. Guidelines were developed and efficient systems for the analysis of epidemiological trends and the monitoring of drug distribution were operational. Imaginative and innovative approaches were devised in order to supplement current leprosy control activities centred on MDT, such as the leprosy elimination campaign (LEC) and the Special Action Projects for the Elimination of Leprosy (SAPEL) [16].

By the end of 2000, the WHO stated that leprosy had been eliminated in 98 countries of the world and announced the creation of a Global Alliance to eliminate leprosy as a public health problem from every country in the world by 2005 [17].

### Global Alliance to Eliminate Leprosy (GAEL) and its setbacks

In 1999, the GAEL was formed to inject new energy into the elimination campaign, bringing together WHO, the governments of major endemic countries, the Japanese Nippon Foundation, the Novartis Foundation for Sustainable Development, DANIDA, and ILEP. Quite soon, major contrasts emerged between some of the GAEL partners, namely between WHO and ILEP, who always remained critical of the elimination -focused strategy. The clash was so strong that ILEP was expelled from the alliance at the end of 2001 [5]. Later on, probably in response to the increasing pressure to achieve leprosy control, WHO invited an independent team of experts to evaluate the GAEL [5]. The evaluation report, published in 2003, recommended that WHO take leprosy activities beyond 2005, dropping the elimination goal in favour of an explicitly broad-based approach to the control of the disease, the avoidance of nerve damage, and the rehabilitation of those in need [10]. The team also explicitly called for the reconstitution of a refined alliance, where collaborators worked more openly, collegially, and inclusively [10]. The process of dialogue and collaboration with WHO headquarters in Geneva was later re-established. WHO worked in collaboration with its partners, ILEP, The Nippon Foundation and Novartis, and developed a new strategy for the period 2006–2010 for sustaining quality leprosy control activities [18]. The WHO has played a global leadership role in this alliance by initiating and helping to conduct leprosy programmes worldwide [19]. The introduction of MDT, the concept of Elimination of Leprosy as a Public Health Problem and continuous technical support was provided by the organization. At the start of the new millennium, the Final Push Strategy was put forward which spelt out special strategies for countries which had achieved and which were yet to achieve elimination. This strategy was also universally accepted by all the endemic countries. The WHO helped in capacity building of general healthcare staff, IEC activities and monitoring at the national and peripheral levels through Leprosy Elimination Monitoring exercises and special programmes like SAPEL and LECs. WHO supports management information systems, logistics and infra-structure. Research, especially operational research is supported widely as are the various national and international conclaves of the project staff [19].

The Nippon Foundation and SMHF have been active worldwide since 1975 in combating leprosy, having spent more than US$ 200 million in this period. The SMHF does not directly implement any leprosy programme but funds projects through the WHO or other ILEP members. From 1995 to 1999, the Foundation provided MDT drugs free of charge to anybody who needed it anywhere on the planet [19]. This Foundation has been working towards ending social discrimination against leprosy patients. Its chairman visited endemic states in India participating in advocacy workshops for the public and for media persons [19]. The Novartis Foundation provided free supply of MDT to some countries like India through the WHO since 2000 and committed to doing so till 2010 [19]. Beginning 1993-94, the World Bank provided support to the Indian National Leprosy Eradication Programme through a soft loan. The first phase of the World Bank supported project was completed in September 2000. It had an impressive report-reducing prevalence from 24/10,000 in 1992 to 3.7/10,000 by March 2001. The project greatly expanded MDT availability, assisted active and continued passive case detection, supported prevention of disability and placed greater emphasis on reducing stigma and increased awareness [19]. As a bilateral agency, DANIDA supported the leprosy programme in many countries including India since 1986 [19].

### Modified Leprosy Elimination Campaign (MLEC)

Leprosy Elimination Campaigns (LEC) were recommended by the WHO for detecting serious leprosy cases- referred to as “cases of consequence”. The proposal was to identify these cases and treat them with MDT. LEC was a combination of three elements: capacity building measures for local health workers to improve MDT services; increased community participation to strengthen elimination activities at peripheral levels; and diagnosing and curing patients, particularly “cases of consequence”. LEC aimed at case detection through passive methods, with individuals coming forward for diagnosis [19]. In India, the MLEC greatly helped in recording hitherto undetected 0.99 million new leprosy cases for treatment within a short period of time [20]. This massive population based exercise resulted in reducing the hidden case load of leprosy, curing patients, averting deformities, reducing transmission potential and increasing self reporting for timely treatment. Health personnel received an exposure to leprosy cases and MDT programme which facilitated integration of the disease treatment services [20]. Leprosy services changed from erstwhile vertically run programmes to integrated services. This increased accessibility of leprosy service to the people nearer to their home on all working days. Large numbers of health staff were trained to make them proficient in suspecting leprosy and providing health education to the patient, family and community members [20]. Repeated mass campaigns helped in increasing public awareness about leprosy, its curability, and MDT availability in health facilities, resulting in improved number of self reporting for diagnosis and treatment. Stigma associated with the disease in the society is gradually diminishing, although we are still a long way to completely remove it. The introduction of a simplified information system (SIS) significantly helped in streamlining data generation, reporting and monitoring of the programme [20].

However, there have been challenges to MLEC in countries like India. Since leprosy programmes were shifted from the hands of specially trained vertical leprosy staff and long time associated NGOs to the general health services staff, some degree of slowness in picking up requiring level of efficiency was observed [20]. This caused certain operational barriers into the programme like wrong diagnosis, re-registration of old cases as new, and poor follow-up of cases for treatment completion. During the integration process, countries were supposed to retain erstwhile vertical staff as a nucleus that should provide technical support to general staff. The process of implementation was however very slow. Although the programme made some efforts to provide the POD services through the general health system through existing NGOs, the same was not adequate to the need of all leprosy patients [20].

### Special Action Project for the Elimination of Leprosy (SAPEL)

Crucial to any public health programme is access to treatment for those who need it most. The WHO suggested SAPEL as an initiative to provide MDT services to patients living in difficult-to-reach areas and neglected population groups. By definition, SAPEL had to be flexible in its implementation strategy and adapted to the needs of the individual community and target audience [19]. For example, in India between 1997 and 2000, 23 SAPEL initiatives were actualized in hilly and geographically inaccessible areas. A population of 320,000 was covered and 153 new cases of leprosy were detected and introduced to MDT. The average cost, per case of detection and treatment, came to Rs 6,500(about=$3) [19] indicating that to treat a case of leprosy is quite cheap. In Brazil, in 2003, the leprosy prevalence was 4.2 cases per 10000 population with a detection rate of 22.3 per 100000 population especially in impoverished states of the north and north east. The highlight of activities included national campaigns on BBC, PAHO/WHO and the health ministry to encourage early help seeking behaviour and to improve awareness of the signs of leprosy. The major constraints to eliminating leprosy in Brazil included limited access to leprosy diagnosis and treatment in endemic areas, non-adherence to fixed duration treatment and a very centralized programme [21].

In Madagascar, the highlight of SAPEL activities included updating of registers and the development of plans to intensity leprosy elimination efforts. The SAPEL constraints in Madagascar were poor geographical accessibility to many health facilities especially in the rainy season and the restructuring of the national leprosy programme [21].

### Leprosy Elimination Monitoring (LEM)

This is a component of case validation that was added to the leprosy elimination strategy. The WHO document: Leprosy Elimination Monitoring Guidelines for Monitors-2000 was used as reference [22, 23]. The purpose of monitoring was to assist decision-makers and programme managers to assess the progress towards leprosy elimination, to make an action plan, implement it and to measure its impact. Monitoring a minimum set of indicators that describes the MDT services served the purpose [22, 23].

Taking into account the epidemiological characteristics of leprosy, and the large number of grey areas in our understanding of the disease, it was important to select carefully the outcome indicators that would be monitored-incidence is the most relevant indicator, but may be the most difficult one. Prevalence, as a composite indicator varies considerably, depending on the operational components of interventions. Incidences of disability and of MB cases in the community could be very useful to evaluate the leprosy situation. The uneven distribution of leprosy, as well as the role of various local factors, calls for caution when extrapolating the results from one place to another. There was a clear need to collect a set of indicators to enable programme managers in endemic countries to analyse the results of their work, increase their motivation and convince the decision-makers [24].

LEM was meant to complement existing information systems and methods for reviewing elimination programmes. The main purpose of LEM was to collect a limited number of indicators that describe the performance of MDT services at the national, sub-national and peripheral levels in endemic countries. The term ‘MDT services’ refers to comprehensive health activities, including: diagnosis, classification, prescription of treatment, delivery of MDT, case-holding and cure of leprosy patients. In some situations, these indicators could help in identifying areas where special campaigns or projects could be implemented [24].

In India, for example, the child proportion, which had decreased in 2003, marginally increased in 2004 [19]. The trend of MB proportion was slowly increasing. The female proportion remained low and stagnant over three years. In 2004, the diagnosis of leprosy was made and treatment initiated in 80% of the health facilities, and MDT services provided on all days in 90%. The median delay in diagnosis, which had declined in 2003, remained stable at seven months in 2004. The MB cure rate was below 85% in 2004. The pauci-bacillary (PB) leprosy cure rate was at 93-94% in all three years [14]. MDT stock management at the health facility level had not made progress in three years. The use of data in calculating essential indicators had improved in the final two years. However, only a third of health facilities had calculated at least three essential indicators. The case validation exercises carried out in 2003 and 2004 in India suggested that the proportion of wrong diagnosis was static at 9.4%; the proportion of re-registration was significantly higher in 2004 (18.7%) than in 2003 (13.5 %). At 12.8%, the proportion of wrong grouping in 2004 was slightly higher than in 2003 (11.2 %) in India [19].

### Cameroon as one of the countries with a successful programme of leprosy elimination

In 1974, Cameroon demonstrated its political will to fight against leprosy by putting in place a national system of co-ordination. The first survey was made in 1975. It recorded 43,816 people with leprosy at a time when the population numbered 5,856,863-that is 75 cases of leprosy per 10,000 inhabitants. Eleven years later, when Aide aux Lépreux Emmaüs-Suisse established a regional office in Yaoundé, there was a need to find ways to develop structures and resources that could ensure greater follow-up of the National Programme [25].

By 1996, the number of people with leprosy had fallen considerably to 1850 among a population of 12,121,002; the equivalent of 1.51 cases per 10,000 inhabitants. This drop reflected the efficiency of the strategies that had been implemented and the introduction of MDT as the means of treating people with leprosy in 1985. Initially only 14.3% of people needing treatment for leprosy received MDT. Today all those who are diagnosed with leprosy in Cameroon are treated with MDT. In 1998, there was less than one person per ten thousand with leprosy [25].

In Essimbiland of North Western Cameroon, leprosy prevalence is high [26, 27]. Boyo and Menchum divisions had leprosy prevalence of 3.35/10,000 and 4.5/10,000 respectively in 1996 [28]. In 2008, these divisions still had the highest leprosy prevalence (Essimbiland with 1.7 cases per 10000 population and Boyo with 2 cases per population) [29]. In 2006, 416 new cases of leprosy were recorded giving a prevalence of 0.3 cases per 10000 and the number in 2007 was 492 giving a prevalence of 0.3 cases per 10000. Besides looking after people who are taking treatment for leprosy, much remains to be done for those individuals who have been cured of this disease, but are still facing high social stigma [25, 30]. One of the key objectives of the current leprosy plan 2008-2011 of the National Leprosy Elimination Programme is to rid Cameroon of leprosy by treating all people who have this disease. Another important aim is to ensure, through early detection, that no other people develop grade two disabilities as a result of leprosy. It is vital to ensure that all people affected by leprosy have access to good health care. Equally, it is necessary that the authorities and donors are aware of the necessity to continue fighting leprosy [25].

### The final push to eliminate Leprosy

The WHO dubbed the ambitious project “the final push to eliminate leprosy”. The strategy behind the slogan involved expanding MDT services to all health facilities and making leprosy diagnosis available, training health workers to diagnose and treat leprosy, promoting leprosy awareness and encouraging people to seek and continue treatment [22]. However, despite the impressive results obtained so far, this is still a work in progress. According to the 2003 WHO report, ten countries in Africa, Asia, and Latin America showed prevalence above the selected threshold [31]. Topping this list was a group of six endemic countries that together account for 83% of the leprosy cases registered worldwide: India, Brazil, Madagascar, Mozambique, Nepal, and Tanzania. According to WHO, in 2004 the number of patients with leprosy worldwide was 457,792 [18]. Worryingly, whereas prevalence figures have fallen steadily in the last two decades, the annual rate of new cases did not follow a comparable trend-this rate has remained essentially unchanged over the past ten years. Indeed, the number of new cases detected during 1994 was 560,646, increasing to 804,357 in 1998, and fell to 513,798 in 2003 [18, 31]. On the basis of available information, the WHO considers the global target of leprosy elimination as reached, and has shifted the strategy to the national level, for which elimination was rescheduled for the end of 2005 [31]. In its plans, the WHO estimated that eight out of the remaining ten countries would reach the new target, while India and Brazil will probably need additional time. The key constraints to eliminating leprosy in those countries that lag behind the elimination campaign vary greatly from country to country. In some leprosy-endemic countries (such as Madagascar, Mozambique, Nepal, and Tanzania), access to many health facilities is extremely poor because of difficult terrain, displacement of populations in remote areas, or for security reasons [31]. In other countries, such as Brazil, important problems arise from the much centralised structure of the leprosy programme, and from its poor integration with general health services [31]. To deal with these very different scenarios, the strategies identified by WHO vary accordingly, proposing in some cases the complete restructuring of the national leprosy programme [31].

### Hurdles to the elimination struggle

Elimination created the unrealistic expectation that the leprosy problem could be solved by the year 2000. In the first place, the elimination goal was not feasible in several areas which had high incidence of leprosy [32]. Even if elimination was to be attained, significant numbers of new cases would continue to occur and many people with physical imperfections, severe psychological, economic and social problems caused by leprosy would need continuous assistance. MDT has been very effective in treating leprosy cases. However, there is no evidence of decline of disease transmission since the inception of MDT [33]. Statements to the effect that MDT can interrupt transmission are unsubstantiated, as there are no convincing data which show this to be true. Evidence-based studies deny the effect of MDT on leprosy transmission [34]. The failure of WHO to acknowledge BCG's major influence on trends in leprosy, combined with the exaggeration of MDT's effects is more a reflection of politics and advocacy than of science [34]. Jakeman et al [35] in Bhutan, reported a marked fall of registered prevalence and case detection rate but no concomitant fall in the proportion of child cases, which indicated that MDT had no effect on leprosy transmission. In Cameroon, some leprosy endemic foci still have high prevalence of child cases [26]. Currently, leprosy researchers do not have the technological means of interrupting transmission reliably. Thus, they depend on measuring disease burden in terms of prevalence figures. This puts them in the unusual situation of being able to foresee the end of leprosy even before its epidemiology is fully understood [36]. Innovative, flexible and more rapid methods will be needed, both to find hidden cases and to implement MDT. Nsagha and collaborators [37] have identified a number of operational barriers to MDT implementation in Cameroon. Diagnostic specificity will need to be improved. Honesty in reporting should be encouraged but projecting prevalence data (by governments) will need to be discouraged, regardless of pressure to produce the desired figures [38]. Leprosy spreads where the inhabitants face unfavourable socio-economic and related conditions: penury, hunger, malnutrition, high infant mortality, unemployment, low minimal salary, inflation, recession, poor housing, slums, promiscuity, lack of hygiene, illiteracy, lack of family planning, ecological degradation, corruption, political instability, rebellion and internal war [39]. These obstacles have not been eliminated in countries endemic with leprosy. The relation of leprosy to poverty needs more attention. Where economic development has occurred leprosy has been reduced. This means that the medical and preventive efforts of the WHO and the countries concerned must be supplemented by efforts in the areas of rehabilitation and economic improvement [40]. Economic development in poor leprosy endemic countries does not take place overnight; it will therefore take many years before leprosy is eliminated from these countries with concurrent economic development. The historical trend of leprosy in Norway [41], Great Britain [42], Europe [43], North America [44], Hawaii [43], South Africa [45], Israel [46], Japan [47] and the Philippines [48] seem to indicate that therapeutic action has not been the single most important factor in leprosy decline. In these countries, the decreasing incidence and prevalence of leprosy has long antecedated the sulphone era beginning in the late 40s of the 20th century. Thus, some other powerful forces must have brought about this decline which cannot be sufficiently explained by a natural leprosy regression. There seem to be strong evidence that socio-economic development has been the dominant factor in the subsidence of endemic leprosy in these countries. The intricate linkage between an improved standard of living and a declining leprosy incidence seems to be well established [49, 50]. This insight, which however is not of recent origin, ought to have serious implications on present and future anti-leprosy campaigns. It should clearly modify the past emphasis placed on policies advocating predominant reliance on physical isolation of leprosy patients and/or on strategies that focus almost exclusively on treatment of those afflicted by the disease [49, 50]. The WHO's reliance on MDT for the elimination of leprosy may be misleading.

Leprologists and people involved in disease control feared that once leprosy is declared eliminated as a public health problem, the future of anti-leprosy services and of leprosy workers and researchers will be at high risk [51, 52]. Elimination is not eradication, and it must be clear that leprosy will continue to exist even in areas where the elimination goal has officially been reached [5]. The term elimination itself makes people think the problem is over, which can have detrimental effects on the future commitment of governments to sustain control activities, making it at the same time difficult for leprosy NGOs and scientists to raise funds for field and lab work [5]. Others believe that the concept of elimination itself, and the choice of prevalence as an indicator to measure the progress of the WHO-orchestrated campaign, is scientifically devoid of significance -as were the 2000 and 2005 deadlines. The wrong indicator (prevalence instead of incidence rate) has been selected to assess the progress toward leprosy elimination [32]. New-case detection and the proportion of children among new cases would serve much better to monitor the real disease status than prevalence [5].

The International Leprosy Association′s Technical Forum has also noted that the expectation that reduction of prevalence to very low levels would lead to a reduction of the incidence within a few years was overoptimistic, as there was little evidence to support this hypothesis [53]. Since patients are only registered while they are on medication, prevalence figures by the WHO standards vary depending on how long treatment lasts. The decrease of prevalence is attributable primarily to the cleaning of the registers (discharge of cured or defaulting patients), to shortening the duration of treatment and, in some countries, to improved diagnostic accuracy, and is not a consequence of reduction of the transmission of *Mycobacterium leprae* [5]. There is probably a lot more leprosy in the world than the World Health Organization currently accepts [3]. The political implications of the elimination goal, and the way it was enforced by the WHO, have also been questioned. Over the last years, the elimination target has more and more become a political target rather than an epidemiological or program quality target [5]. The prevalence of patients registered for treatment has become the goal in itself, and the actual goal-reduction of the leprosy transmission and incidence has practically got out of sight [5]. Furthermore, the fixing of numerical targets may put excessive pressure on national leprosy programme managers, discouraging them from actively working to detect new cases, which in turn could jeopardise the country′s elimination status [5]. The presence of extra-human reservoirs of leprosy, the lack of an effective anti-leprosy vaccine, cultural taboos, and concomitant improvement in the standard of living in countries endemic with leprosy seems to be pertinent issues in leprosy research. In the past, slogans for the elimination of malaria and tuberculosis have all been failures to the extent that these diseases are serious threats to human health nowadays [54]. The WHO elimination strategy has to be reconsidered. When questions about the control of a disease are unanswered, we must conclude that research, contrary to current opinion is more necessary than ever [55]. Prevalence figures were used to measure progress, and the number of patients with leprosy decreased from an estimated 12 million in 1985 to 0.6 million in 2002 [31, 56]. Disease prevalence is measured by counting all patients receiving treatment at a defined moment and expressing this as a ratio using the population as the denominator. Prevalence figures are therefore affected by operational aspects of programmes, such as the length of treatment; for example, halving the duration of treatment for patients receiving MB-MDT from 24 months to 12 months halves the prevalence figures for that group. Additionally, the means of administration may also affect the numbers; for example, patients receiving single-dose treatment (rifampicin, ofloxacin and minocycline) for single skin lesions do not appear in prevalence figures nor do patients who received their 6-month course of PB-MDT early in the calendar year since only patients registered on 31 December are counted for that year [1]. Using WHO′s definition, South Africa attained elimination in 1924 but new leprosy cases continue to be detected in the northern Transvaal [57]. Preventing patients with nerve damage from progressing to disability and deformity is a challenge that will last for the patient′s lifetime. Patients with anaesthesia and muscle weakness need to be taught how to care for their hands and feet. Specialist footwear needs to be provided for patients with deformities of their feet to prevent ulceration. Ulcer management forms a large part of any leprosy service. Staff should work with patients to prevent ulceration from recurring by identifying the cause of the initial injury. Preventing disability is critical to the success of a programme. We need to understand the routes that lead to disability [1].

## Conclusion

The evidence base available to inform whether leprosy is being eliminated is highly positive based on the availability of MDT blister packs. There are concerns that leprosy was not the right disease to be targeted for elimination as there are no reliable diagnostic tests to detect subclinical infection including the lack of a vaccine. Social stigma to leprosy is still very high for both new cases and those discharged from treatments that are deformed. The high social stigma affects rehabilitation of patients because their original communities are not willing to accept them. Furthermore, in some communities, people are not willing to buy goods produced by lepers from their rehabilitation trades. Leprosy affects mostly the poor but the relationship between poverty and the disease needs to be established. The use of prevalence as an indicator to assess elimination instead of incidence rate is misleading and needs to be revisited. Leprosy relapses and the prevention of disabilities among patients on treatment and discharged cases are still major problems. Humans are not the only reservoirs of M. leprae as extra-human reservoirs have been identified in monkeys and armadillos. There is an increase in the burden of child cases indicating that transmission is ongoing. MDT treats leprosy very well but there is no proof that it concurrently interrupts transmission and this relationship can be investigated further through more research. There is need for more research in the areas that are hurdles to the elimination strategy in order to better understand the epidemiology of leprosy and eradicate it from the world.

### The way forward For Leprosy elimination and eradication

In order to ensure that leprosy does not go underground, the elimination strategy must be swiftly converted to a post-elimination strategy [51]. This new focus should be on integrating leprosy control activities into primary health care services, assuring early case detection, adequate chemotherapy, prevention of disability for all patients with nerve damage, and physical rehabilitation of those already disabled [1, 51, 58]. Work to dispel the social stigma of leprosy and to introduce patients back into their communities (rehabilitation) must also be strengthened, in order to end social discrimination toward people with leprosy [1]. Leprosy remains a disease of the poor, although the exact social factors that put people at risk have not been identified [59]. To break this link between leprosy and poverty, the disease should be included in the portfolio of diseases associated with poverty, and leprosy work incorporated into poverty reduction programmes [60]. Lockwood and Suneetha [1] have suggested that the routine use of vaccination could benefit the outcome of WHO′s anti-leprosy strategy. Although the development of a specific and highly effective vaccine against leprosy is not available, the tuberculosis vaccine (Bacillus Calmette-Guérin), has proved to offer some immunity to leprosy. Its reported efficacy ranges from 34% to 80% in different countries [4]. Despite this variable efficacy, bacillus Calmette-Guérin vaccination is already widely used in leprosy-endemic countries. Who should be vaccinated, when, and how often in order to achieve maximal protection among the population are all a matter for debate [61]. Another promising intervention for leprosy prevention is from an Indonesian study, conducted on five islands, which found that giving people who are in close contact with leprosy patients a short course of rifampicin can reduce their risk of developing the disease [62]. Important epidemiological research issues remain to be addressed including developing improved diagnostic tests and better ways to monitor and treat nerve damage, and understanding why MDT has not interrupted transmission [1]. We shall not be able to eliminate leprosy until we have a better understanding of its natural reservoir [5]. There are several interesting studies that indicate that there may be an environmental reservoir for M. leprae -perhaps even in the soil in endemic regions [63]. Healthy human carriers of M. leprae do exist, and it has been found that sensitive molecular methods can detect the DNA from M. leprae in people who showed non-specific skin lesions and who had not been thought to have leprosy. Even after a ten-year MDT programme, more than 5% of healthy individuals in a leprosy-endemic area were positive for M. leprae DNA in their nasal passages, suggesting a high level of environmental contamination [63]. Before we can talk about eliminating leprosy, we have to understand where the organisms are found and the circumstances resulting in an active infection [3].We cannot be talking of eliminating leprosy when its epidemiology is not well understood.

Data for evaluating the impact of MDT on transmission are not readily available because leprosy has a long incubation period [64]. Mathematical modelling of the transmission and control of leprosy showed that the elimination strategy reduces transmission slowly, with a predicted annual decline in incidence ranging from 2% to 12%. Future projections of the global leprosy burden indicated that 5 million new cases would arise between 2000 and 2020, and that in 2020 there would be 1 million people with WHO grade 2 disability [64]. To attain the elimination of leprosy, it is necessary to find effective interventions to interrupt transmission of M. leprae and practical diagnostic tools to detect levels of infection that can lead to transmission. This requires extensive research in the areas of epidemiology and microbiology [64].
